# Refined variant calling pipeline on RNA-seq data of breast cancer cell lines without matched-normal samples

**DOI:** 10.1186/s13104-025-07140-3

**Published:** 2025-02-15

**Authors:** Sonja Eberth, Julia Koblitz, Laura Steenpaß, Claudia Pommerenke

**Affiliations:** 1 Human and Animal Cell Lines, Leibniz-Institute DSMZ-DSMZ-German Collection of Microorganisms and Cell Cultures GmbH, Inhoffenstraße 7B, 38124 Braunschweig, Germany; 2 Bioinformatics, IT and Databases, Leibniz-Institute DSMZ-DSMZ-German Collection of Microorganisms and Cell Cultures GmbH, Inhoffenstraße 7B, 38124 Braunschweig, Germany; 3 Zoological Institute, Technische Universität Braunschweig, 38106 Braunschweig, Germany

**Keywords:** Variant calling, RNA-seq, Breast cancer, Cancer cell lines, COSMIC, DSMZCellDive

## Abstract

**Objective:**

RNA-seq delivers valuable insights both to transcriptional patterns and mutational landscapes for transcribed genes. However, as tumour cell lines frequently lack their matched-normal counterpart, variant calling without the paired normal sample is still challenging. In order to exclude variants of common genetic variation without a matched-normal control, filtering strategies need to be developed to identify tumour relevant variants in cell lines.

**Results:**

Here, variants of 29 breast cancer cell lines were called on RNA-seq data via HaplotypeCaller. Low read depth sites, RNA-edit sites, and low complexity regions in coding regions were excluded. Common variants were filtered using 1000 genomes, gnomAD, and dbSNP data. Starting from hundred thousands of single nucleotide variants and small insertions and deletions, about thousand variants remained after filtering for each sample. Extracted variants were validated against the Catalogue of Somatic Mutations in Cancer (COSMIC) for 10 cell lines included in both data sets. Approximately half of the COSMIC variants were successfully called. Importantly, missing variants could mainly be attributed to sites with low read depth. Moreover, filtered variants also included all 10 cancer gene census COSMIC variants, a condensed hallmark variant set.

**Supplementary Information:**

The online version contains supplementary material available at 10.1186/s13104-025-07140-3.

## Introduction

Cell lines are well accepted for studying complex biological processes and testing therapeutic efficacies of new agents, while contamination and misidentification of cell lines have caused massive costs and irreproducible research [1]. Hence their authentication and molecular characterisation are required for selection of appropriate *in vitro* models. For proper model selection in breast cancer research, the mutational landscape in breast cancer relevant genes should be considered for both inherited germline and somatic mutations in recurrently mutated genes (e.g. *BRCA2, TP53*), which might harbour tumour drivers [2, 3].

Commonly, genomic sequencing data is applied for identifying single nucleotide variants (SNVs) and small insertions and deletions (InDels) from whole exome and whole genome sequencing (WES/WGS). However, variants on expressed genes can be extracted from RNA-sequencing (RNA-seq) data as a byproduct from transcriptional profiling [4–6]. It is used as diagnostic tool [7], for cell line identification [8] or for studying genetic heterogeneity in cell line populations [9].

On the other side, identifying variants without matched-normal samples, frequently absent for cell lines, adjustment for confounding common germline variants is required [10–13], otherwise leads to unreliable, biased, and inflated variant prediction [14–16]. Decontamination of germline variants usually occurs by filtering out common variants. To date, filtering variants on tumour-only samples on RNA-seq basis still remains to be optimised.

This study proposes a straightforward pipeline to enhance variant filtering on RNA-seq without matched-normal pairs for breast cancer cell lines as described with modifications [17]. As there was no suitable pipeline existent for this dataset, we developed an adjusted workflow on variants called via the HaplotypeCaller from the popular Genome Analysis Toolkit (GATK) [18], that showed higher sensitivity compared to Mutect2 [19] and was employed by the Cancer Cell Line Encyclopedia (CCLE) [20]. Here, several filter steps are proposed, which haven’t been specified for tumour-only RNA-seq data of cell lines to this extent so far.

## Materials and methods

### Cell lines and RNA-seq

All authenticated 29 human breast cancer cell lines are available at the DSMZ cell line bank (Germany) [17]. RNA-sequencing and analysis were performed as described [17, 21]. Expression data were made accessible via DSMZCellDive [22]. Library sizes spanned between 30-60 million 150bp paired-end reads for each sample, which was described to suffice for calling variants robustly on RNA-seq data in tumour samples [23]. For gaining non-redundant reads for variant calling, insert sizes were aimed at 2x150bp length [24]; here, average mapped read lengths varied around 298bp. Raw data are stored at BioStudies (S-BSST1200) and at ArrayExpress (E-MTAB-14655).

### Variant calling pipeline

**Fig. 1:**
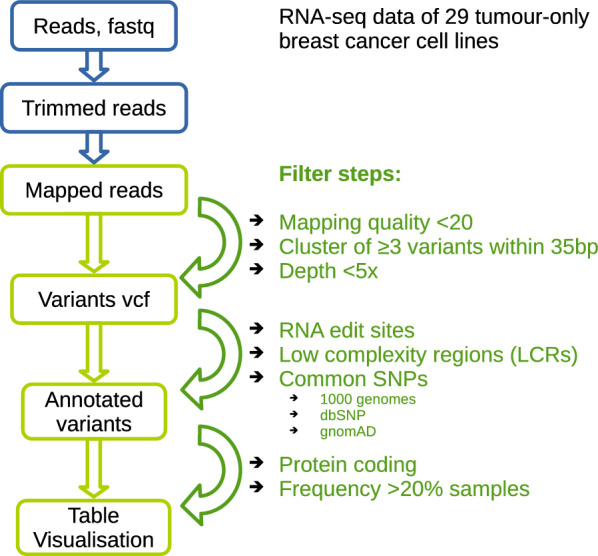
Scheme for filter steps in the presented pipeline based on RNA-seq tumour-only breast cancer cell lines. As the majority of germline variants are dispensable and cannot be detected without matched-normal samples, several filter steps were applied to the identified variants. Detailed descriptions are given in the text

SNVs and small InDels were called on RNA-seq basis as described previously [17] with altered filtering steps: (a) an added low complexity regions (LCRs) filter, (b) a 1/3 frequency filter in the sample set, which was set to 20% in this study, and (c) the omitted PolyPhen/Sift filter, as silent variants were contained in the COSMIC evaluation comparison. An overview on the filtering steps is given in Fig. 1.

Specifically after trimming and mapping (see Supplementary Methods), variants were called by the GATK HaplotypeCaller (4.3.0) following best practices [25], including variants with a minimum mapping quality threshold for variant calling of 20, and omitting variants with <5 read depth and clusters of three or more variants in windows of 35 bp were applied by using the GATK tool bundle [25].

Regions within RNA-edit sites from REDIportal [26] and within LCRs [27, 28] were excluded for variant detection due to quality reasons by applying vcftools (0.1.16) [29] and SnpSift (5.1d) [30].

For filtering common variants, data from 1000 genomes project phase3 [31], gnomAD r2.1.1 [32], and dbSNP v156 [33] were implemented setting the allele frequency to >0.01 using SnpSift, snpEff [34], vcftools, vcf2maf [35], and VEP (105) [36] while concentrating on coding regions.

In addition, variants occurring in more than 20% of the samples were removed, since many of these variants were located in homopolymer or repetitive regions.

Open access to this pipeline is availbale at zenodo [37] and github [38], variant data at the European Variation Archive (EVA) [39] (PRJEB82834).

### Variant evaluation

Extracted variants were compared to COSMIC data (v97) [40] with cell line source DSMZ and labeled with verified or known (see Supplementary Table S1). Analysis of sensitivity and specificity were based on these congruent 400 variants and 10 cell lines (see Supplementary Fig. S1).

Beside automatically generated COSMIC variants, COSMIC CGC variants were matched, derived from expert-curated cancer mutant census (CMC, v98) [41].

Visualisation of highly mutated genes was done as waterfall plot with the R package GenVisR (1.30.0) [42].

## Results

### Variant calling

SNVs and InDels were called on the RNA-seq data of 29 breast cancer cell lines without matched-normal samples, which we have recently characterised [17]. Lacking normal pairs for variant calling caused flooding with numerous insignificant variants, but kept potentially inherited pathogenic germline variants, e.g. in *BRCA2*, and demanded a profound filtering.

**Fig. 2:**
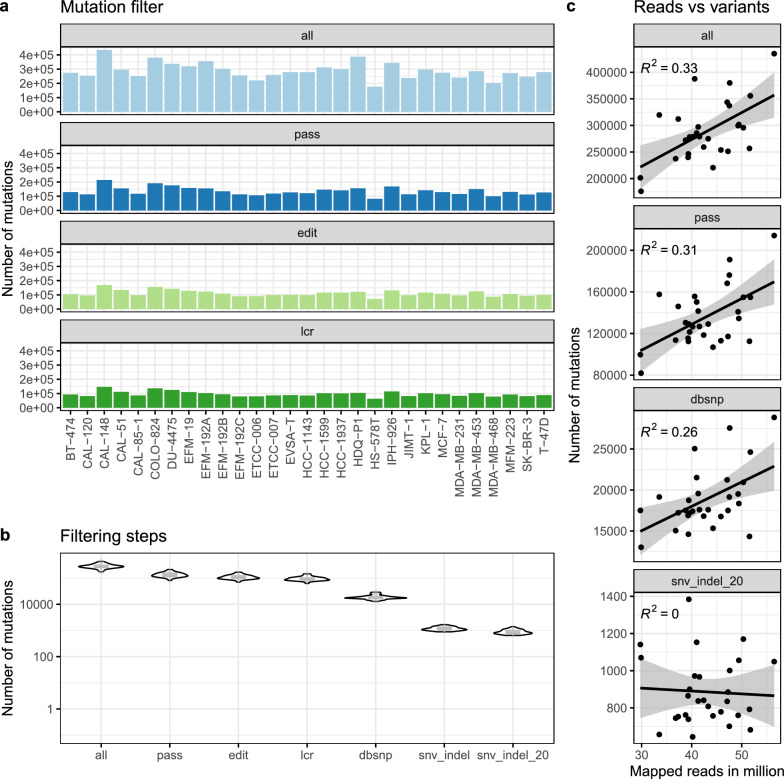
Amount of variants for all 29 breast cancer cell lines based on RNA-seq data. **a** Filtering by read depth and quality (pass) reduced variant numbers markedly, whereas RNA-edit sites (edit) and low complexity regions (lcr) affected less variants. **b** Further filtering of dbSNP (dbsnp) data lowered numbers per sample substantially. Finally, focussing on variants in protein coding regions (snv_indel) and variants in less than 20% of samples resulted in about 1000 variants per sample (snv_indel_20). Mutations included single nucleotide variants (SNVs), insertion and deletions (InDel). **c** Correlation between mapped million reads and filtered variants decreased with every filtering step

Filtering out low quality sites by mapping quality and read depth halved the number of variants with initial sizes of about 176,000–435,000 (Fig. 2a). Mutations were excluded, if they were located within known posttranscriptional RNA-editing sites (31–46% of all variants), which may result in a modified nucleotide on RNA level but does not relate to variants on DNA level [43], and within low complexity regions (27-39%), where sequencing is less accurate [28]. Further removal of common variants by dbSNP reduced the number of variants to the level of 5-9% of initially called variants (Fig. 2a). After excluding all common variants by 1000 Genomes, dbSNP, gnomAD curated data, as well as variants outside protein coding regions, about 1000 variants per sample remained (Fig. 2b, snv_indel).

Sequencing errors for RNA-seq have proved to bias SNVs and InDel detection in repetitive regions [44, 45]. Since we found identical variants for many cell lines residing within homopolymers or repetitive regions, an additional filter on variants in >20% of cell lines was applied (Fig. 2b, snv_indel_20), resulting in an additional reduction (17-33%). Finally, 0.2$-$0.6% of all variants remained ranging from 644 to 1384 per sample (see Supplementary Table S2). Remarkably, the weak correlation between library sizes and called variants diminished with each filtering step (Fig. 2c).

### Variant evaluation

**Fig. 3:**
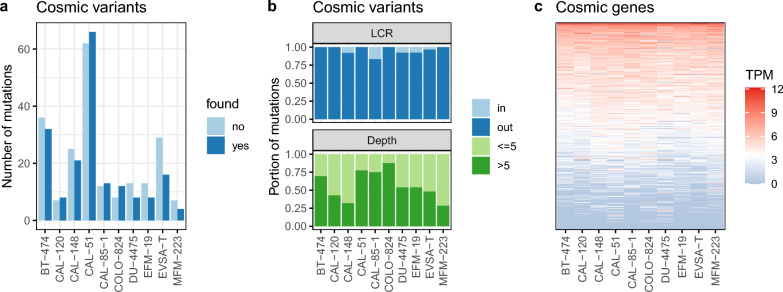
Comparing filtered variants called from RNA-seq with genomic COSMIC variants. **a** 10 breast cancer cell lines were of the same origin as employed in this study. 188 of the 400 verified COSMIC variants in the 10 cell lines were recognised by our pipeline (found: yes). **b** A few missed variants were attributed to low complexity regions (LCR), whereas a great portion of missed variants fell out due to low depth (Depth). **c** Expression of the 353 genes associated with the 400 COSMIC variants was illustrated as heatmap adding 1 on transcripts per million (TPM) values prior to log2 conversion

In order to investigate accuracy, filtered variants were compared to COSMIC data generated by genome sequencing. Of 1020 cancer cell lines included in COSMIC, 10 were authenticated breast cancer cell lines also originating from the DSMZ culture collection and could be used for evaluation. A total of 400 verified COSMIC variants in 353 genes of these 10 cell lines served as basis for comparison, of which 188 could be determined by our workflow (Fig. 3a, Supplementary Table S1). While sensitivity remained unchanged, specificity increased over the filtering procedure (Supplementary Fig. S1). Most of the missed variants could be traced to low read depth (<5) and some were filtered out due to LCR localisation (Fig. 3b). Two variants identified by the pipeline were discarded by the last filter frequency across all breast cancer samples (>20%) resulting in 212 missed COSMIC variants (Supplementary Table S1). Analysis of the 353 genes as transcripts per million (TPM) revealed that (~60%) of the genes were expressed <16 TPM (Fig. 3c, Supplementary Table S3). This is in agreement to a study, in which over 65% variants in coding regions were missed by RNA-seq over WES due to low expression [46].

Apart from the automatically generated COSMIC variant list, a further plausibility check was to compare the filtered variants to the COSMIC cancer gene census (CGC) representing expert-curated cancer-driving gene data. CGC derived mutation census comprised 10 variants for *PIK3CA*, *PTEN*, *APC*, and *TP53* in seven breast cancer cell lines, which were identified by our pipeline (Table 1).

**Table 1:** Cancer gene census (CGC) variants in all COSMIC variants from DSMZ breast cancer cell lines

Cell line	Gene symbol	HGVSG	Mutation AA	Mutation description
BT-474	*PIK3CA*	3:g.179199158G C	p.K111N	Substitution - Missense
CAL-148	*PTEN*	10:g.87957976T A	p.I253N	Substitution - Missense
CAL-148	*PTEN*	10:g.87960984C T	p.Q298	Substitution - Nonsense
CAL-148	*PIK3CA*	3:g.179234297A G	p.H1047R	Substitution - Missense
CAL-51	*PIK3CA*	3:g.179218294G A	p.E542K	Substitution - Missense
DU-4475	*APC*	5:g.112840323G T	p.E1577	Substitution - Nonsense
EFM-19	*PIK3CA*	3:g.179234297A T	p.H1047L	Substitution - Missense
EVSA-T	*PTEN*	10:g.87961047_87961050del	p.T319	Deletion - Frameshift
EVSA-T	*TP53*	17:g.7674241G C	p.S241C	Substitution - Missense
MFM-223	*PIK3CA*	3:g.179234297A G	p.H1047R	Substitution - Missense

All 10 CGC variants of the overlapping CGC breast cancer cell line variants were found by our pipeline. HGVSG and Mutation AA follow the Human Genome Variation Society (HGVS) nomenclature standard for genomic and protein/amino acid levels, respectively

Additionally, a summary of the extracted variants was visualised (Supplementary Fig. S2). For this, variants of all 29 breast cancer cell lines were restricted to the 353 genes of the COSMIC variants and filtered to the mutation types as listed in Supplementary Fig. S2a. The topmost 50 genes with highest number of variants were selected. The gene on rank one was the tumour suppressor *TP53*, on rank five *PIK3CA* and rank six *BRCA2*, all implicated in breast cancer progression [2, 3, 47]. The major fraction of variants harboured missense mutations (Supplementary Fig. S2). Functional effects of specific mutations were addressed previously [17].

## Discussion

Some limitations of RNA-seq based variant calling are tissue specific variability, depth of coverage and consequentially allelic drop-out events, RNA-editing [5, 26], or sequencing artefacts [44, 45]. Nevertheless, the two last points can be addressed by filter adjustment. Additionally, RNA-seq can be exploited twofold for transcriptomics and genetic variation. Moreover, RNA-seq was found to reveal potential new somatic variants over WES [5]. Tumour mutational burden (TMB) detected by RNA-seq was shown to resemble the TMB determined on genomic data [12].

In this workflow of variant detection on RNA-seq data of breast cancer cell lines without matched-normal samples, we strived for variants including germline ones, since inherited risk factors are well-known in recurrently mutated genes for breast cancer [2, 3]. Since variant calling including germline variants results in massive variant amounts, we included following downstream filters: coverage depth, RNA-editing sites, LCRs, three different common variant databases, and sample frequency for coding regions, of which parts only were applied elsewhere for RNA-seq based variant calling on patient data [48]. Concerning RNA-editing sites, about 16 millions of A-to-I events, which are sequenced as guanosine, are described for humans [26]. As RNA-edits cannot be distinguished from genomic variants by RNA-Seq, variant calls at those were excluded. According to Li, 2014 [28], LCRs comprise 2% of the human genome, in which the majority of SNVs and InDels are called with false positive rates of 10-40%, arguing for a further filter. As we observed the same variants in >20% of cell lines, residing in homopolymer and repetitive sequences, variants detected across more than one fifth of samples were omitted. Although 7% of breast cancer patients were predicted to carry inherited cancer mutations [49], we cannot rule out that these 29 cell lines fully represent this tumour entity, because some subtypes might be over- or underrepresented in the *in vitro* models. Moreover, for different cancer types this filter needs to be adapted accordingly, e.g. hotspot mutations would be missed by this such as BRAF V600E, found in 35% melanoma patients [50], and specific genes were recurrently mutated in 20% DLBCL patients [51], requiring a higher threshold.

Several workflows on variant detection based on RNA-seq were described, however, for somatic mutations the standard approach includes matched-normal samples [52–54], which are often unavailable for standard cell lines. Among the tools and pipelines, which cope with tumour-only samples, some lack filtering of RNA-edit sites, LCRs [5, 8, 48, 55–57] and common SNPs described in 1000 Genomes, gnomAD, and dbSNP [58, 59], which are part of our pipeline, or lack open source code [60]. More recently, machine or deep learning tools for classifying and filtering variants have been designed to serve a broad range of different cancer types [4, 12].

Finally, while working with cell lines, it is inevitable to ensure their authenticity, since differences between laboratories have been observed due to contamination and misidentification [1, 61]. Here, the combination of authenticated cell lines and their molecular characteristics warranted quality to the 29 breast cancer cell lines. It adds methological aspects to our recent publication on these mentioned tumour cell lines [17]. This provides comprehensive and novel insights to a variety of models to study breast cancer for development of new therapies.

## Limitations

Neglected aspects of this study critical for estimating relevance and potential pathogenicity of the extracted variants:

- Failing relevant variants occuring at high frequency within certain populations
- Copy number alterations
- Abnormal zygosity
- Manual adaptations to adjust specific data sets and cancer types
- WES/WGS for resolution of allelic drop-outs and lowly expressed genes

## Supplementary Information

Supplementary material 1.

Supplementary material 2.

Supplementary material 3.

Supplementary material 4.

## Data Availability

Raw fastq files are stored at BioStudies (S-BSST1200) and at ArrayExpress (E-MTAB-14655) associated with ENA (PRJEB83077). Gene expression data can be accessed freely at DSMZCellDive (https://celldive.dsmz.de/rna/breast-cancer, released 6 Feb 2024). Workflow and scripts are archived at zenodo (DOI:10.5281/zenodo.13759327) and github (https://github.com/claupomm/RNA-seq_snv_tumour_only, accessed 10 Jan 2025). The variant data for this study are deposited in the European Variation Archive (EVA) at EMBL-EBI (PRJEB82834).

## Abbreviations

ADAR: Double-stranded RNA-specific adenosine deaminase
CCLE: Cancer cell line encyclopedia
CGC: Cancer gene census
CMC: Cancer mutant census
COSMIC: Catalogue of somatic mutations in cancer
GATK: Genome analysis toolkit
EVA: European variation archive
HGVS: Human genome variation Society
InDels: Insertions and deletions
LCRs: Low complexity regions
SBS: Single base substitutions
SNVs: Single nucleotide variants
TMB: Tumour mutational burden
TPM: Transcripts per million
WES: Whole exome sequencing
WGS: Whole genome sequencing
