# Supplementary material for: Refined variant calling pipeline on RNA-seq data of breast cancer cell lines without matched-normal samples
Source: BMC Res Notes. 2025 Feb 15;18:67. doi: 10.1186/s13104-025-07140-3 (PMC11829467; doi:10.1186/s13104-025-07140-3)

Supplementary information

Supplementary Methods: Trimming, mapping and calling variants
After trimming via fastp (0.23.4) [1] sequences were mapped via STAR (2.7.10b) on human gencode (42) in two-pass mode [2], and read group adding, read duplicate removal, splitting reads with N in cigar, base recalibration, variant calling by GATK HaplotypeCaller (4.3.0) including variants with a minimum mapping quality threshold for variant calling of 20, and omitting variants with <5 read depth and clusters of three or more variants in windows of 35 bp were applied by using the GATK tool bundle [3].

[1] Chen, S. Ultrafast one-pass FASTQ data preprocessing, quality control, and deduplication using fastp. iMeta 2(2):e107 (2023). https://doi.org/10.1002/imt2.107
[2] Dobin, A. et~al. STAR: ultrafast universal RNA-seq aligner. Bioinformatics 29(1):15--21 (2013). \urlprefix\url{https://doi.org/10.1093/bioinformatics/bts635}
[3] der Auwera, G. A. & O'Connor B. D. Genomics in the Cloud: Using Docker, GATK, and WDL in Terra, 1st edn. O'Reilly Media (2020), URL https://www.oreilly.com/library/view/genomics-in-the/9781491975183/

Supplementary Table S1: Verified COSMIC variants found for the 10 overlapping DSMZ human cell lines for 353 genes specifying which variant was detected after indicated filter steps. Filters include Depth (threshold 5 reads), RNA-edit (in RNA edit sites: no/yes), LCR (within the region: in/out), dbSNP (described common SNP: no/yes), after Depth/RNA-edit/LCR/dbSNP/gnomAD/1000Genomes extraction and Coding (if in coding regions: no/yes), and Frequency (variant samples cutoff <=20%). Additionally, CGC indicates whether corresponding variant is contained in the CGC (no/yes) and was expressed more than one transcripts per million (TPM) read (Expression: <=1 TPM/ >1 TPM).

Supplementary Table S2: Called and filtered variants via the outlined pipeline of this work for 29 breast cancer cell lines. After preprocessing steps and variant calling, subsequent filtering processes included Depth/RNA-edit/LCR/dbSNP/gnomAD/1000Genomes extraction. About a thousand variants remained for each sample.

Supplementary Table S3: Gene expression for the 353 COSMIC variants genes found for the 10 overlapping DSMZ human cell lines in transcripts per million (TPM). Gene expression data can be accessed and visualised interactively at DSMZCellDive (https://celldive.dsmz.de/rna/breast-cancer).

Supplementary Figure S1: (a) Sensitivity, (b) Specificity, and (c) precision based on the overlapping DSMZ-COSMIC variant set for the single filter steps. While variant sensitivity kept nearly constant over the filtering procedure for the 10 breast cancer cell lines, specificity increased as the numerous non-informative called variants were extensively reduced.

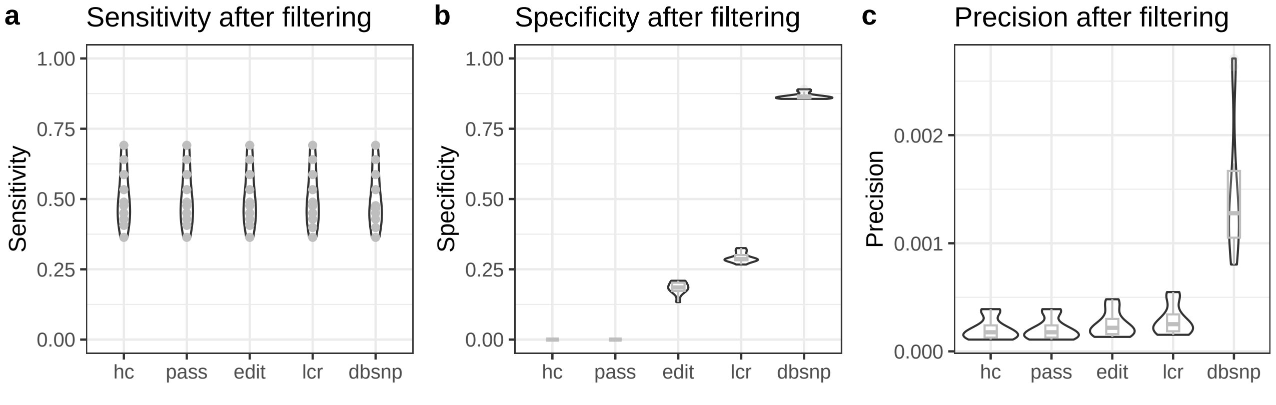

Supplementary Figure S2: Genes with the highest mutational burden as waterfall plot. (a) Of the top50 COSMIC genes with the upmost mutation numbers on rank one TP53 and on rank six BRCA2 were identified. Here, variants were restricted to the mutation types as listed in the legend. (b) The distribution of variant types for the COSMIC gene set as barplot.

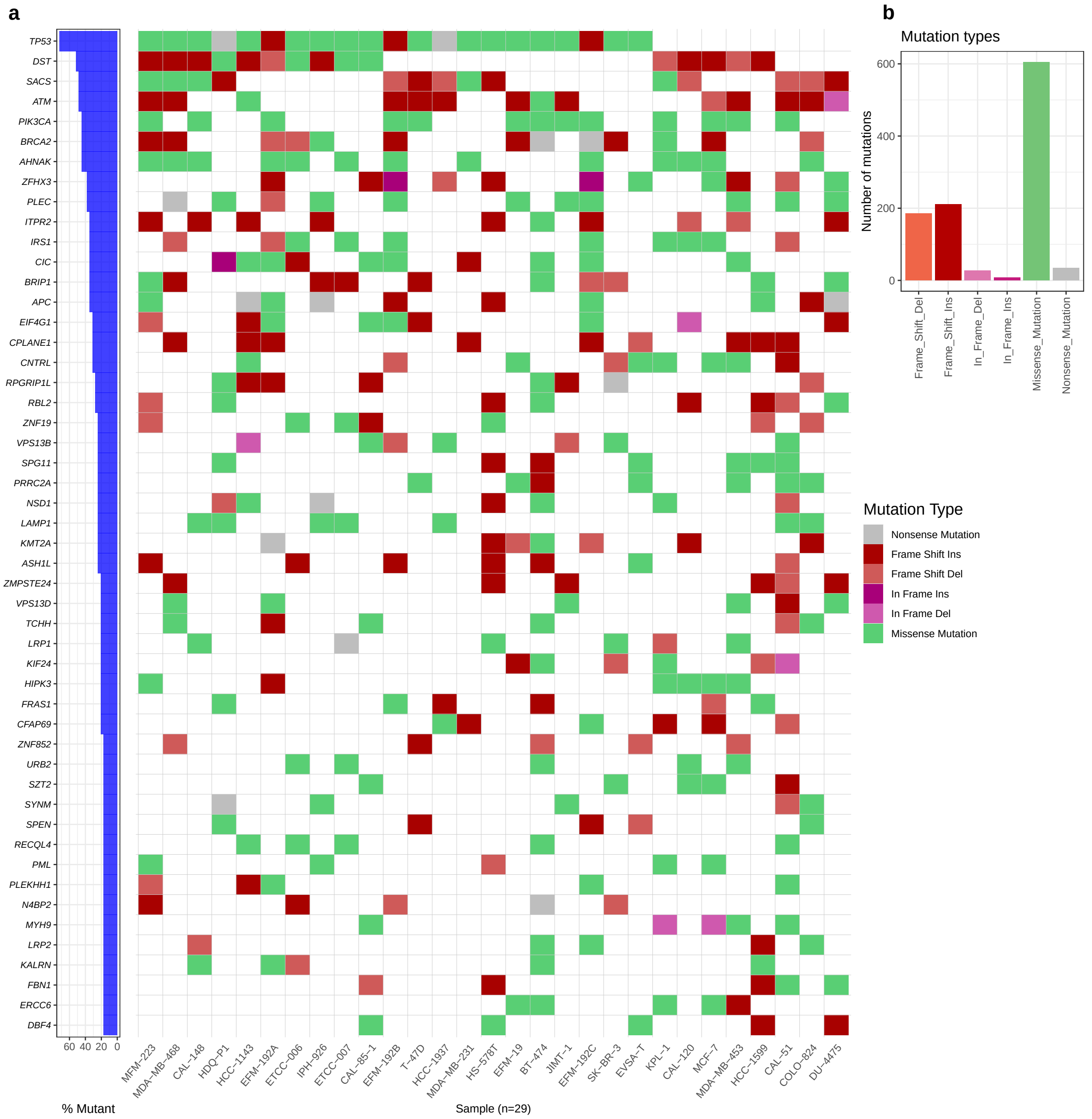

Supplement: Supplementary file 1 — Supplementary material 1. [file 13104_2025_7140_MOESM1_ESM.pdf]
